# Microneedle patch as a new platform to effectively deliver inactivated polio vaccine and inactivated rotavirus vaccine

**DOI:** 10.1038/s41541-022-00443-7

**Published:** 2022-02-28

**Authors:** Sung-Sil Moon, Marly Richter-Roche, Theresa K. Resch, Yuhuan Wang, Kimberly R. Foytich, Houping Wang, Bernardo A. Mainou, Winston Pewin, Jeongwoo Lee, Sebastien Henry, Devin V. McAllister, Baoming Jiang

**Affiliations:** 1 Division of Viral Diseases, Centers for Disease Control and Prevention, Atlanta, GA USA; 2 Micron Biomedical, Inc., Atlanta, GA USA

**Keywords:** Vaccines, Viral infection

## Abstract

We recently reported a lack of interference between inactivated rotavirus vaccine (IRV) and inactivated poliovirus vaccine (IPV) and their potential dose sparing when the two vaccines were administered intramuscularly either in combination or standalone in rats and guinea pigs. In the present study, we optimized the formulations of both vaccines and investigated the feasibility of manufacturing a combined IRV-IPV dissolving microneedle patch (dMNP), assessing its compatibility and immunogenicity in rats. Our results showed that IRV delivered by dMNP alone or in combination with IPV induced similar levels of RV-specific IgG and neutralizing antibody. Likewise, IPV delivered by dMNP alone or in combination with IRV induced comparable levels of neutralizing antibody of poliovirus types 1, 2, and 3. We further demonstrated high stability of IRV-dMNP at 5, 25, and 40 °C and IPV-dMNP at 5 and 25 °C, and found that three doses of IRV or IPV when co-administered at a quarter dose was as potent as a full target dose in inducing neutralizing antibodies against corresponding rotavirus or poliovirus. We conclude that IRV-IPV dMNP did not interfere with each other in triggering an immunologic response and were highly immunogenic in rats. Our findings support the further development of this innovative approach to deliver a novel combination vaccine against rotavirus and poliovirus in children throughout the world.

## Introduction

Rotavirus (RV) and poliovirus (PV) are two enteric pathogens causing severe diarrhea and poliomyelitis and paralysis, respectively, among young children throughout the world^1–4^. Live oral RV vaccine (ORV) and oral PV vaccine (OPV) have been effective in preventing disease, but both vaccines have underperformed in low- and middle-income countries (LMICs) compared with developed countries^5–7^. In addition, both oral vaccines are associated with severe adverse events: rare intussusception in children who received ORV and rare vaccine-associated paralytic polio (VAPP) and circulating vaccine-derived polioviruses (cVDPV) in OPV-vaccinated children. To eliminate the risks of VAPP and cVDPV, a transition program to gradually replace OPV with IPV is under way and will result in the discontinuation of OPV in the coming years when wild polioviruses and cVDPV no longer circulate^8,9^. To overcome the safety and efficacy concerns of ORV, next-generation non-replicating parenteral vaccines are under development^10^. An inactivated RV vaccine (IRV), when formulated with alum gel and administered by intramuscular (IM) injection, proved to be highly immunogenic and protective against an oral challenge with a virulent human strain in animal studies^11,12^.

Currently, most pediatric vaccines are administered by IM injection, which requires a large cold chain storage capacity, needs administration by trained professionals, and often causes pain at the injection site^13^. To improve current vaccine administration, microneedle patches (MNPs) are under development to deliver vaccines to the skin without using hypodermic needles^14^. MNPs have been evaluated to administer a number of vaccines including influenza, IPV, IRV, human papillomavirus (HPV), measles, human immunodeficiency virus (HIV), and hepatitis B virus (HBV) in preclinical studies^15–24^. IPV delivered by dMNP had improved immunogenicity in rhesus macaques^25^ and dMNP-administered IRV induced mucosal immunity and showed a dose-sparing effect in mice^24^. Influenza vaccine administered using a dMNP was found safe and as immunogenic as traditional IM administration among adults in phase I clinical trial^26^.

As new and improved vaccines are considered for expanded programs of immunization (EPI) in countries throughout the world, children face increasingly crowded immunization schedules in the first couple of years in life. Consequently, various combinations of vaccines are under development. A combined IRV and IPV administered by IM injection was found highly immunogenic with no interference between the two vaccines in rats^27^. In the present study, we optimized formulations and processes to fabricate a dMNP for the co-administration of IRV and IPV, examined the immunogenicity of standalone IRV or IPV, and combined IRV-IPV in rats by skin vaccination, and evaluated the long-term stability of IRV and IPV dMNP in vitro.

## Results

### Fabrication and stability of dMNP

**Fig. 1:**
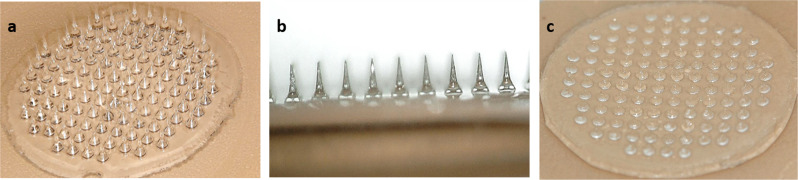
Fabrication of dissolving microneedle patch (dMNP). First-generation dMNP was composed of 112 microneedles (**a**), each MN measuring 700 µm in length, occupying an ~0.8 cm^2^ area mounted on an adhesive backing (**b**). Microneedles were dissolved after insertion into the skin of a rat (**c**).

The present study described the fabrication of a first-generation dMNP with 112 MNs containing carboxymethylcellulose (CMC) and an improved, denser second-generation dMNP with 163 MNs containing methylcellulose (MC). Both first- and second-generation MN arrays occupied an ~0.8 cm^2^ area mounted on an adhesive backing (Fig. 1). The dMNP were solid conical structures made of water-soluble excipients with antigens contained substantially in the tip of the microneedles. For IRV and IPV dMNP fabrication, each dMNP contained 25% more antigen than the target dose based on the delivery rate from a previous study^24^.

**Fig. 2:**
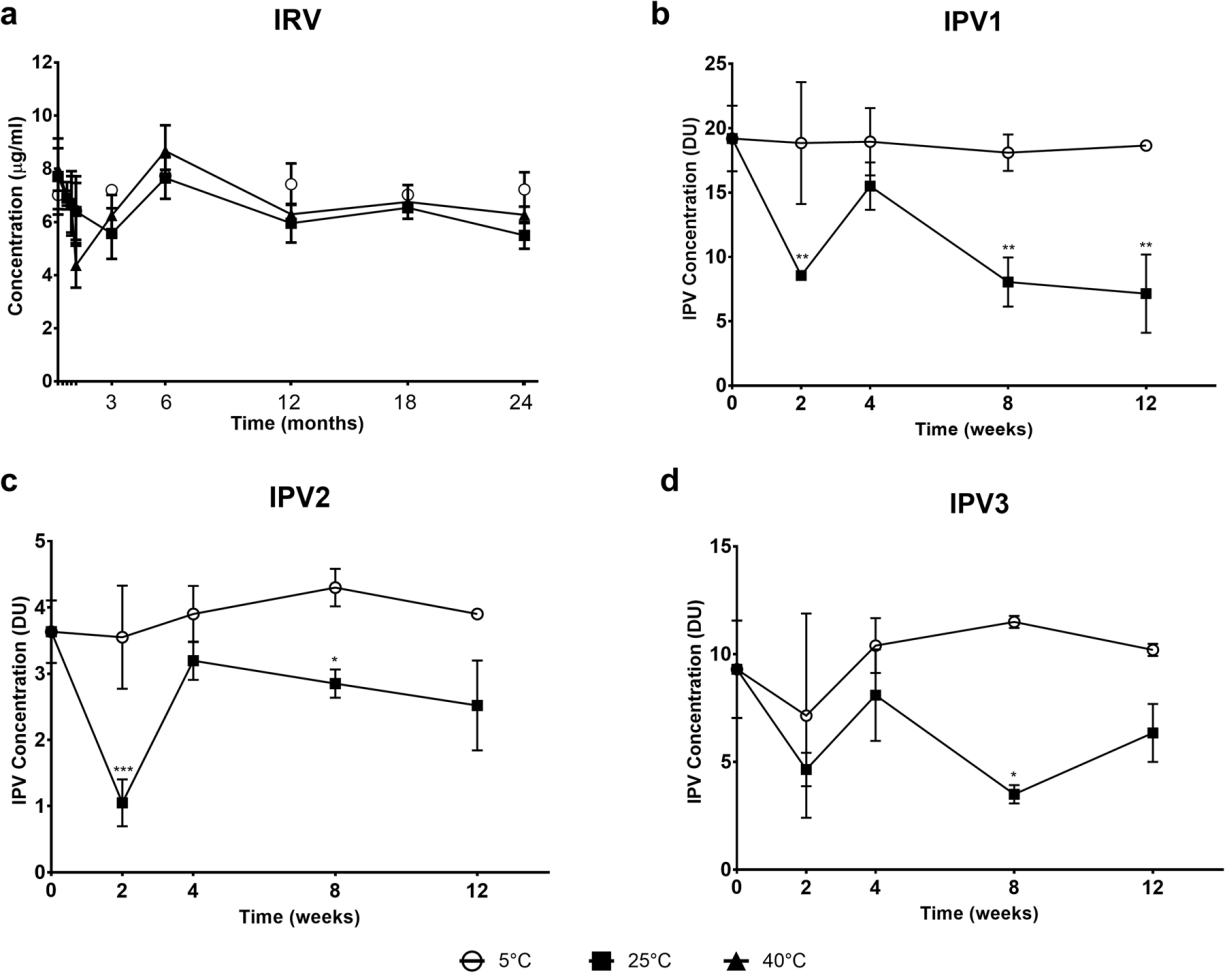
Stability of IRV and IPV on dMNP. Stability of first-generation IRV dMNP stored at 5, 25, or 40 °C up to 2 year (**a**) and first- generation IPV dMNP stored at 5 and 25 °C up to 3 months (**b**–**d**). dMNP patches were analyzed using in-house ELISA for IRV and IPV type 1, 2, and 3 at various time points. Data represent means and standard deviations from three replicate dMNP. Significant differences of data at various time points from baseline concentrations were analyzed by two-way ANOVA and Sidak’s multiple comparison test. * *p* < 0.05, ** *p* < 0.01, ***p < 0.001.

Time-dependent stability tests were performed on first-generation IRV dMNP stored at 5 °C, 25 °C or 40 °C for up to 24 months and IPV dMNP stored 5 °C or 25 °C for 3 months (Fig. 2). IRV dMNP were found to retain RV potency, as measured by VP7 antigenicity, for 24 months under all conditions examined (Fig. 2a). IPV dMNP maintained potency up to 3 months at 5 °C but showed decreased activity at 25 °C after 4 weeks (Fig. 2b–d). dMNP were monitored for appearance; all appeared clear to slightly off white, with correct physical form, and passed appearance tests up to 24 months. Studies to assess the stability of second-generation IRV-IPV dMNP are in progress.

### Delivery efficiency study in rats

**Fig. 3:**
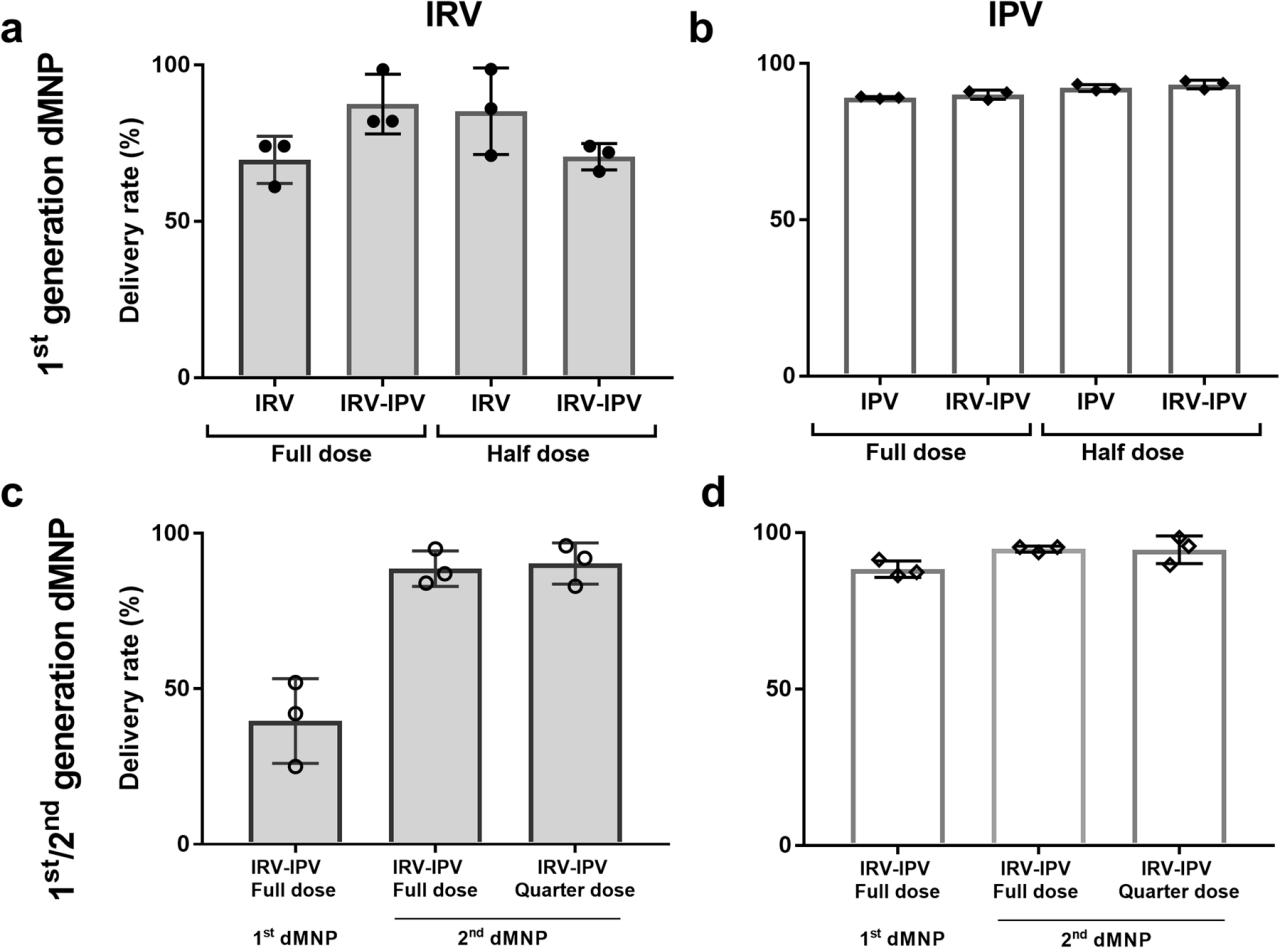
Delivery efficiency of IRV, IPV and IRV-IPV by dMNP in rats. Delivery rates of IRV-IPV by first-generation dMNP (**a**, **b**) and second-generation dMNP (**c**, **d**). First-generation IRV dMNP were produced to deliver 5 µg and 2.5 µg as full and half dose, respectively (**a**). First-generation IPV dMNP were produced to deliver full (40, 8, and 32 DU) and half (20, 4, and 16 DU) human dose of IPV 1, 2, and 3, respectively (b). Second-generation IRV dMNP were fabricated to deliver 5 and 1.25 µg of IRV and full and quarter doses of IPV. Value of antigen delivered was calculated by measuring the residual RV and PV antigen amount on the dMNP after all three vaccinations [(initial dose−residual dose)/initial dose × 100%].

The dMNPs were applied with thumb pressure for 1 min to the back of rats and left in place for 15 min. After application, the inserted tips of the MNs dissolved (Fig. 1c). Measurements of residual IRV and IPV antigens in the inserted first-generation dMNP showed that the rate of dose delivery [(initial dose−residual dose)/initial dose × 100%] to the skin ranged 61–99% for full- or half-dose IRV in standalone IRV or IRV-IPV dMNP (Fig. 3a). The rate of delivery was 81–97% for full- or half-dose IPV in standalone IPV or IPV-IRV dMNP (Fig. 3b).

Because solutions containing CMC cannot be sterilized by filtration and the delivery efficiency of IRV observed in CMC-containing formulations was relatively low and inconsistent, we assessed MC-containing formulations for potential improvement as MC-containing process solutions can be sterilized by filtration. After inoculation, we estimated the delivery efficiency of a full-dose IRV-IPV in second-generation combination dMNP was 83–96% and 93–98%, respectively (Fig. 3c, d). By contrast, delivery efficiency was 25–52% for IRV and 82–95% for IPV in the full-dose IRV-IPV first-generation dMNP. Both IRV and IPV second-generation dMNP showed improved delivery versus first-generation dMNP. Similar delivery efficiency was observed in the quarter or full-dose IRV-IPV second-generation dMNP.

### IRV dose range study in rats

**Fig. 4:**
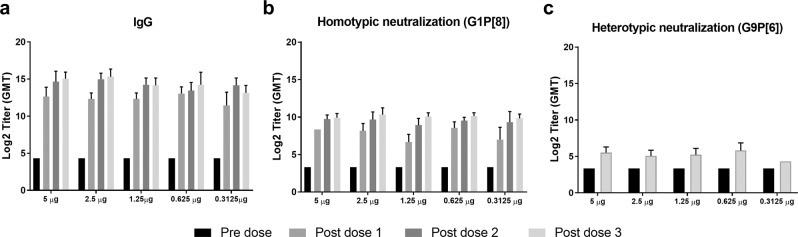
IRV dose range study of the first-generation dMNP in rats. Animals (*n* = 6) were immunized with IRV dMNP three times (day 0, 21 and 42) at five different doses (5, 2.5, 1.25, 0.625, and 0.3125 µg). Sera were collected before the first, second, and third dMNP dose (days 0, 21, 42) and after third dose (day 63), and tested for RV-specific IgG (**a**) and neutralizing activity against a homotypic strain, Wa (G1P[8]) (**b**) or a heterotypic strain, CDC-6 (G9P[6]) (**c**). Serum specimens were tested for IgG at an initial dilution of 1:100 and if negative, a value of 20 was assigned for determining GMT and illustration. Neutralizing activity was tested at an initial dilution of 1:20. Statistical analysis was carried out to compare antibody titers at pre-dose 1 and post-dose 1, 2, or 3 in animals that received full IRV dose and four fractional IRV doses using two-way ANOVA with Bonferroni multiple comparisons test. No statistical significance were observed.

To determine the dose required to elicit effective antibody response, we tested first-generation IRV dMNP at 5, 2.5, 1.25, 0.625, and 0.3125 µg per dose in rats (Fig. 4). IRV delivered by dMNP induced strong and elevated titers (1600–25,600) of RV-specific IgG at post-dose 1 for all doses tested. IgG titers further increased following doses 2 and 3, although not all increases were significant (Fig. 4a). Rats that received 5, 2.5, 1.25, and 0.625 µg of IRV developed high levels of neutralizing antibodies (NA) against the homotypic RV Wa (G1P[8]) strain largely in a dose-dependent manner (post-dose 1 titer: 80–640; post-dose 2: 320–2560; post-dose 3: 320–2560) (Fig. 4b). These vaccinated rats also developed elevated but lower levels of cross NA against the heterotypic RV CDC-6 (G9P[6]) strain post-dose 3 (titer 20–160) (Fig. 4c). No statistical significance was observed in antibody titers at pre-dose 1 and post-dose 1, 2, or 3 in animals that received full IRV dose and four fractional IRV doses.

### IRV-IPV co-administration study in rats

**Fig. 5:**
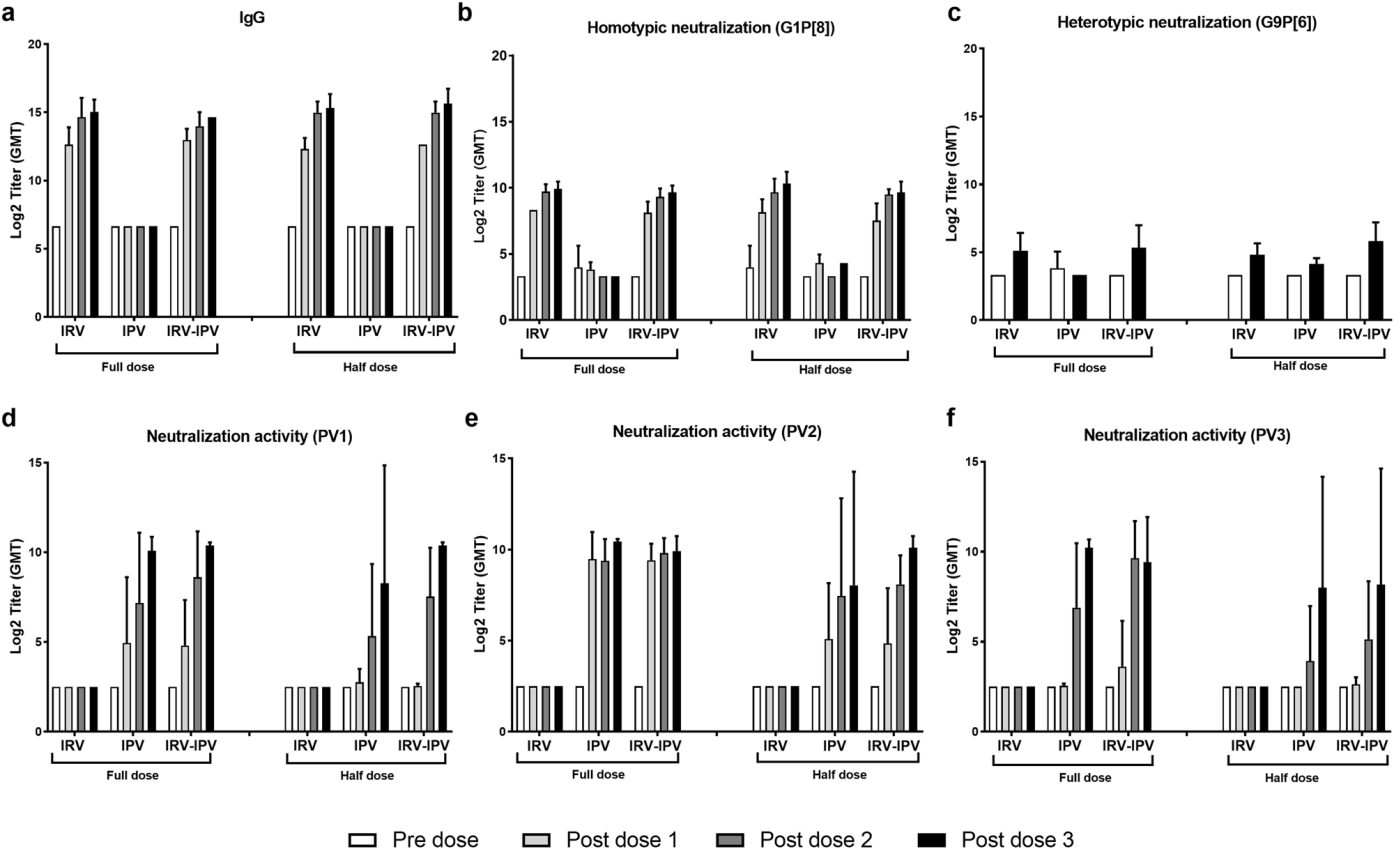
Lack of interference between IRV and IPV delivered by the first-generation dMNP in rats. Sera were collected before the first, second, and third dose (day 0, 21, 42) and after the third dose (day 63) and were tested for RV-specific IgG (**a**) and neutralizing activity against a homotypic strain (**b**) or a heterotypic strain (**c**), or PV-specific neutralizing antibody to type 1 (**d**), 2 (**e**) and 3 (**f**) as described in the text. Statistical analysis was done to compare antibody titers in animals that received standalone IRV or IPV and combined IRV-IPV at pre-dose 1 and post-dose 1, 2, or 3 using two-way ANOVA with Bonferroni multiple comparisons test. No statistical significances were observed. Data are presented as geometric mean titers ± 3 SD for each group (*n* = 6).

To examine whether co-administration of IRV and IPV in one dMNP resulted in any vaccine interference, we compared the immunogenicity of the two vaccines at two dose levels administered alone in individual patches or together in a combined patch in rats (Fig. 5). We found that a full or half dose of standalone IRV or IRV-IPV induced similar elevation of RV-specific IgG titers (1600–25,600 for full dose and 1600–6400 for half dose) and NA titers (160–640 for full dose and 80–640 for half dose) against the homotypic Wa strain after the first vaccination; antibody titers increased at post-dose 2 and remained elevated at similar levels up to 63 days (Fig. 5a, b). We also observed elevated titers of NA (20–320) against the heterotypic CDC-6 strain after 3 full or half doses of vaccines administered alone or together (Fig. 5c). The differences in antibody titer between post-vaccine doses 1 to 3 of full dose standalone IRV and full IRV-IPV were not statistically significant. Similarly, the differences in antibody titer between post-vaccine doses 1–3 among full dose IRV or IRV-IPV and half-dose IRV or IRV-IPV were not statistically significant.

Two or three doses of standalone IPV or combined IRV-IPV in the first-generation dMNP at full or half dose induced protective titers (≥1:8) of NA to the corresponding PV types 1, 2, and 3 in rats (Fig. 5d–f). The differences in antibody titer between post-vaccine doses 2 and 3 of full dose standalone IPV and full IRV-IPV were not statistically significant. Similarly, the differences in antibody titer between post-vaccine doses 2 and 3 of full-dose IPV, full-dose IRV-IPV, and half-dose IPV or half-dose IRV-IPV were not statistically significant. Of the three IPV types, IPV 2 was the most immunogenic and induced high geometric mean titers (GMTs) to PV type 2 even with a single dose.

**Fig. 6:**
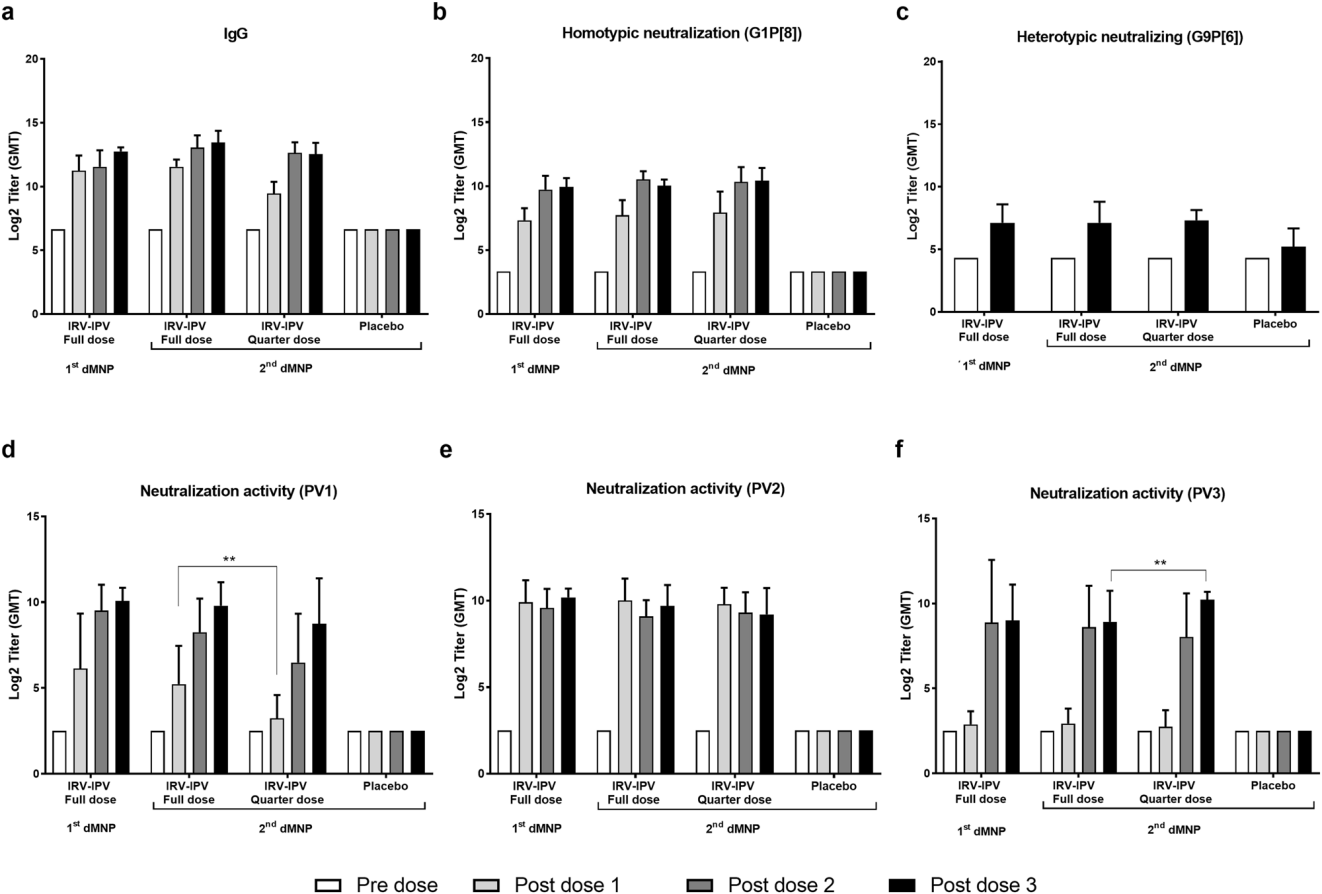
Antigen dose sparing of the second-generation IRV-IPV dMNP in rats. Sera were collected before the first, second, and third IRV-IPV dMNP dose (day 0, 21, 42) and after the third dMNP dose (day 63) and were tested for RV-specific IgG titer (**a**) and neutralizing activity against a homotypic strain (**b**) or a heterotypic strain (**c**), or PV-specific neutralizing antibody to type 1 (**d**), 2 (**e**) and 3 (**f**) as described in the text. Statistical analysis was done to compare antibody titers in animals that received standalone IRV or IPV and combined IRV-IPV at pre-dose 1 and post-dose 1, 2, or 3 using two-way ANOVA with Bonferroni multiple comparisons test. **p < 0.001. Data are presented as geometric mean titers ± 3 SD for each group (*n* = 10).

To determine whether the second-generation IRV-IPV dMNPs were as immunogenic as the first-generation IRV-IPV dMNP, rats were vaccinated three times with a full dose of the combination vaccine. Three full doses of the second-generation IRV-IPV dMNP induced higher titers of IgG and NA to the homotypic RV Wa strain than those from first-generation IRV-IPV dMNP though differences were not statistically significant (Fig. 6a, b). Three full doses of first- and second-generation IRV-IPV dMNP induced lower but similar levels of NA to a heterotypic CDC-6 strain (Fig. 6c). A full dose of first- or second-generation IPV-IRV dMNP induced equivalent levels of protective NA titers to PV types 1, 2, and 3 in a dose-dependent manner except for type 2 (Fig. 6d–f). Rats that received placebo second-generation dMNP had no detectable antibody titers to RV and PV throughout the study. Of note, three full doses or quarter doses of the second-generation IRV-IPV dMNP induced similar titers of RV-specific IgG and RV- or PV-specific NA post doses 1–3 in rats. At least two doses of IRV or IPV were needed to induce high antibody titers with the exception that the prime dose for IPV type 2 was as potent as that from three doses.

## Discussion

We recently reported that IRV and IPV when formulated together with aluminum adjuvant and administered intramuscularly did not interfere with each other and showed dose sparing potential in rats and guinea pigs^27^. In the present study, we optimized conditions for manufacturing a combined IRV-IPV dMNP and found that this IRV-IPV dMNP administered to skin was highly effective in inducing a robust antibody response in rats. This potent immunogenicity, measured by IgG and NA response, was similar whether IRV and IPV were administered alone or in combination, again indicating no interference between the two vaccines and establishing the proof of concept for skin immunization against rotavirus and poliovirus with a novel combination vaccine using a dMNP.

We demonstrated equivalent antibody titers to PV or RV when rats received a full or quarter dose of IPV and IRV in combination delivered using a second-generation dMNP and thus showed the potential for significant antigen sparing for skin delivery of both vaccines. In addition, rats developed similar levels of RV-specific IgG and homotypic NA titers when IRV in the range of 0.3125–5 µg was administered, but higher dose (≥0.625 µg) of IRV was required to stimulate a heterotypic NA. Our findings of apparent IRV dose sparing agree with those of an early study in which a fractional (20%) dose of IRV dMNP was as effective as a full dose in inducing serum antibody response and the expression of the gut homing receptor LPAM-1 on T and B cells in spleen and mesenteric lymph nodes of vaccinated mice^24^. However, in contrast to our current results, previous studies did not show dose sparing for IPV dMNP in macaques^25^. The reasons for this discrepancy are not known. Nevertheless, our findings of antigen sparing for all three IPV types are significant as there are only a handful Salk IPV manufacturers and currently there is a supply shortage worldwide^28^. Demonstration of apparent antigen dose sparing of both IRV and IPV, together with adjuvant sparing seen with IRV dMNP^23,24^, should help alleviate potential cost and safety concerns of this novel combination vaccine.

The Global Polio Eradication Initiative has relied entirely on oral poliovirus vaccines (OPVs) to eliminate wild-type polioviruses throughout the world. However, circulating vaccine-derived polioviruses (cVDPV) which are mutated from the original OPV strains are associated with outbreaks of poliomyelitis in some settings of low vaccine coverage. Consequently, IPV has been recently added as part of the strategies to eradicate PV globally. IPV as a booster dose by IM injection has been shown to induce intestinal immunity in children who are primed with OPV and help achieve polio eradication in India^29^. Since IPV is given as a standalone vaccine in many developing countries, it requires a separate cold chain and intramuscular administration by trained healthcare workers, causes pain and produces medical waste. In addition, current IPV is relatively expensive compared to other EPI vaccines (e.g., DTwP-Hib-HBV)^30^. A combined IRV-IPV for skin immunization using a dMNP may not encounter the same challenges and would simplify immunization programs with potential benefits in low- and middle-income countries^31^. Moreover, a recent World Health Organization Advisory Panel recommended that after OPV withdraw worldwide, all countries should implement a 2-dose IPV schedule in routine immunization schedule, the first dose at or after 14 weeks and the second dose ≥4 months later (e.g., with the first MMR vaccine), administered at full or fractional doses^32^. A combined IRV-IPV dMNP at the recommended IPV schedule would protect against a disease that may not exist in the majority of countries and sustain global polio eradication. On the other hand, this new dMNP combination vaccine could boost intestinal immunity to RV in children who are primed with ORV as demonstrated by the sustained intestinal immunity from IPV booster dose among children who received at least 5 doses of trivalent OPV in India^29^ and thus would extend protection against severe diarrhea in the second year and beyond among children. The immunogenicity and efficacy of ORV are lower in the first year of life and further declined in the second year in many low- and middle-income countries (LIMCS)^33^, and additional oral booster doses have not shown any real meaningful effect in those children^34–36^.

The present study is subject to some limitations and implications. We previously conducted 3 side-by-side studies to compare immune response and protective efficacy of IRV administered by IM injection and by skin vaccination using a hollow microneedle device in piglets and a solid or dissolving MNP in mice^23,24,37^. We demonstrated comparable or slightly superior protective immunity with skin immunization over IM administration in all three studies, thus we did not include an IM arm in the present study. We evaluated two formulations and found that the second generation of dMNP was more efficient to deliver IRV and IPV than the first generation. It is known that CMC-containing solutions have high viscosity, make it more difficult to dry the antigen into the tips of the dMNs, and generally result in lower and more variable delivery efficiency in the first generation dMNP. In addition, the second generation dMNP contains almost 50% more MNs and when filled with MC-containing solution, there should be more antigen towards the tips of the MNs. We demonstrated high delivery efficiency (>80% for IRV and >90% for IPV) for full or quarter dose of combined IRV-IPV by subtracting the quantity of antigen remaining in the unused patch from the total using pig skin. Future studies are needed to quantify the loss, including residual antigen left on the surface of the skin during MNP administration. We tested small volumes of serum samples from individual mice for neutralizing antibodies against three PV strains and one homotypic RV strain as well as RV-specific IgG. We only had small volumes left and had to test pooled samples in each group against one heterotypic RV strain. Of note, we optimized conditions and found we needed to use slightly higher titer of the heterotypic G9P[6] strain for optimal performance of the assay. Future studies will need to assess whether lower titers of heterotypic neutralizing antibody are effective against non-G1P [8] strains and examine if cross-reactive IgG can serve as a correlate of protection against infection and disease in clinical trials among children.

In conclusion, this study is the first to show that a combined IRV-IPV dMNP is highly stable, immunogenic and well-tolerated in rats. Our findings support moving the IRV dMNP into IND-enabling studies (e.g., GMP manufacturing, GLP-toxicology study) and subsequently a phase 1 clinical trial in healthy adults. We will then design follow-up studies to support the clinical development of this novel IRV-IPV dMNP for safety, immunogenicity, and efficacy in children.

## Methods

### Vaccine preparation

CDC-9, a human G1P[8] RV strain, was cultivated in Vero cells and triple-layered particles (TLPs) and double-layer particles (DLPs) were purified from cell supernatants by using CsCl gradient centrifugation^38^. The ratio of TLPs and DLPs was approximately 9:1 as measured by protein concentration with a Bradford assay. TLPs and DLPs in 50 mM HEPES, 150 mM NaCl, 5 mM CaCl_2_ [pH 7.2-7.5] supplemented with 7% D-sorbitol were inactivated at 62 °C for 6 h and protein concentration was determined by Pierce™ Coomassie Bradford protein assay kit (Thermo Fisher Scientific, IL, USA) before being used for the fabrication of dMNP. Mono-bulks containing approximately 900, 450, and 650 DU/ml of IPV types 1, 2, and 3, were donated by GlaxoSmithKline, and were concentrated approximately 150, 100, and 75-fold by volume, respectively, and suspended in 0.375 mM histidine buffer (J.T. Baker^®^, PA, USA). Mono-bulk concentration and buffer exchange were performed using Amicon Ultra centrifuge spin filters with 100 kDa MW cutoff at 4 °C, 4000 × *g* (Millipore Sigma, MA, USA). D-antigen contents were determined by ELISA^39^.

### Dissolving microneedle patch (dMNP) fabrication

Dissolving microneedle patches were fabricated from polydimethylsiloxane (Dow Corning, MI, USA) molds using a two-step casting method (i.e., first an antigen-containing solution followed by a polymer matrix solution).

First-generation dMNP consisted of 112 700-µm-tall microneedles (MNs) with a total MN volume of 1.8 µl. For the fabrication of IRV dMNP to deliver 5 μg IRV, the antigen casting solution had a concentration of 1.13 mg/ml IRV, 5% *w/v* sucrose (VWR, OH, USA) and 1% *w/v* sodium carboxymethylcellulose (CMC, Spectrum, CA, USA) in 50 mM HEPES, pH 7.3 (Amresco, OH, USA), 150 mM NaCl and 5 mM CaCl_2_ (VWR) buffer. For the fabrication of IPV dMNP to deliver 40, 8, and 32 of IPV types 1, 2, and 3, respectively, the casting solution had a concentration of 12.5, 2.5, and 10.0 DU/μl, 3.75% *w/v* maltodextrin (Sigma-Aldrich, MO, USA) and 1.25% *w/v* xylitol (Alfa Aesar, MA, USA) in 0.375 mM histidine buffer. First-generation combination patches were generated from IRV and IPV dMNP manufactured at 20% more than the target dose, which was sectioned in half and assembled on an adhesive backing. Combination patches had a target delivery dose of 5 μg IRV and 40, 8, and 32 DU of IPV types 1, 2, and 3, respectively. To manufacture dMNP at lower doses, solutions were prepared by diluting higher antigen concentration solutions, such as the antigen casting solution used for the full dose combination patch described above, with excipient solution to keep excipient concentration constant while decreasing vaccine dose. Antigen solutions were cast onto molds under vacuum of ~27 inHg. The molds were then dried for an hour (4 °C, 3000 × *g*).

Polymer matrix solutions, composed of maltodextrin, xylitol, sodium carboxymethylcellulose in histidine buffer for IPV MNs and polyvinyl alcohol (EMD Millipore, MA, USA) and sucrose in HEPES, NaCl, and CaCl_2_ buffer for IRV MNs, were deposited to form the base of the MN arrays. The IRV MN arrays were dried at 35 °C overnight; IPV MN arrays were dried refrigerated for 2 days. Once dried, an adhesive backing was adhered to the base of the MN arrays and peeled from the mold. All patches were packaged in foil pouches with a 5-g silica gel desiccant sachet.

To improve manufacturing and delivery efficiency, second-generation dMNP, composed of 163 700-µm-tall MNs with a total MN volume of 2.7 µl, were fabricated similarly with the following differences. For the IRV-containing dMNP, the CMC in the formulation was replaced with methylcellulose (MC) and CMC was removed from the polymer matrix solution of IPV-containing dMNP. Since CMC is unable to be sterilized by filtration, methylcellulose (MC) was evaluated in combination with the other excipients and found to be a suitable replacement for CMC in the formulation to be more compatible with future GMP manufacturing. In addition, the combination IRV-IPV dMNP were fabricated as a single patch and not assembled halves of two separate arrays, as done with the first-generation combination dMNP.

Antigen casting solutions for second-generation dMNP were prepared at lower vaccine concentrations while keeping the ratio of vaccine to excipient constant. To fabricate full dose IRV dMNP, casting solutions had a concentration of 0.625 mg/ml IRV, 2% *w/v* sucrose (VWR, OH, USA) and 0.4% *w/v* sodium methylcellulose. IPV casting solutions had a concentration of 5.8, 1.1, and 5.2 DU/μl of types 1, 2, and 3, respectively, 1.5% *w/v* maltodextrin (Sigma-Aldrich, MO, USA) and 0.5 % *w/v* xylitol. Full-dose combination patches were prepared to deliver 5 μg IRV and 40, 8, and 32 DU of IPV types 1, 2, and 3, respectively. Solutions for manufacturing the quarter dose dMNP contained lower vaccine doses while keeping the excipient concentrations constant. After deposition of the polymer matrix solution, all MN arrays were dried at 35 °C overnight.

### Stability studies

First-generation IRV-dMNP to deliver 5 µg of IRV were fabricated and stored at 5, 25 or 40 °C for up to 24 months, whereas IPV-dMNP to deliver 20, 4, and 16 DU (half the commercial dose) of IPV types 1, 2, and 3, respectively were fabricated and stored at 5 or 25 °C for up to 12 weeks. IPV-dMNP stability at 40 °C was not evaluated. The dMNP were tested for IRV or IPV potency by ELISA using a rotavirus VP7-specific monoclonal antibody or monoclonal antibodies to poliovirus types 1, 2, and 3^25,40^. All dMNP were monitored for appearance (e.g., specified number of MNs, free of debris, appropriate shape, and color) throughout the stability studies.

### Animal studies

The immunogenicity of standalone IRV or IPV or combined IRV-IPV dMNP was evaluated in two experiments using four weeks old female Wistar rats (Charles River Laboratories, MA, USA). Animals were divided into 10 groups of 6 rats each to test the first-generation dMNP (Table 1). Rats were anesthetized using 1-2% isoflurane for the vaccination and blood collection. The backs of the rats were shaved with electric shears, followed by the application of a depilatory cream (Nair, NJ, USA) one day before MN patch application. As baseline assessment, pre-vaccination blood was taken from the submandibular vein on the same day of hair removal. Rats were vaccinated with first-generation dMNP to deliver 5 (full dose), 2.5 (half), 1.25 (quarter), 0.625 (eighth) and 0.3125 (sixteenth) µg of IRV for dose range study, or full or half doses of IPV alone or combined IRV-IPV. In a follow-up experiment, rats in 3 groups of 10 each were vaccinated with first- or second-generation full dose IRV-IPV dMNP or second-generation quarter dose IRV-IPV dMNP IRV-IPV (Table 1). Patches were applied with thumb pressure for 1 min on the backs of the rats. After 15 min, the patches were removed. IRV patches were reconstituted in 1 ml of HBSS and IPV patches were reconstituted in 2 ml of blocking buffer to determine the residual antigen amount and dose delivered by RV VP7 ELISA or PV ELISA. Control rats received placebo dMNP in the same manner.

**Table 1:** Targeted delivery dose of IRV, IPV and IRV-IPV dMNP in rats.

Vaccine dosage	First-generation dMNP*	Second-generation dMNP*
Full	IRV: 5 µg	
	IPV: 40/8/32 DU	
	IRV: 5 µg; IPV: 40/8/32 DU	IRV: 5 µg; IPV: 40/8/32 DU
Half	IRV: 2.5 µg	
	IPV: 20/4/16 DU	
	IRV: 2.5 µg; IPV: 20/4/16 DU	
Quarter	IRV: 1.25 µg	IRV: 1.25 µg; IPV: 10/2/8 DU
Eighth	IRV: 0.625 µg	
Sixteenth	IRV: 0.3125 µg	
None		Placebo

Vaccine dosage referred to established human dose for commercial IPV. For IRV, we determined 5, 2.5, and 1.25 µg by Bradford assay as full, half, and quarter dose, respectively, in this study. DU:D antigen unit. * All dMNP incorporated 20% excess antigen(s) to account for a delivery efficiency of ~80%.

All groups received three doses of vaccine separated by 3 weeks. After week 9 (63 days), the rats were euthanized with isoflurane (3–5%). Blood samples were collected at baseline and three weeks after each vaccination. All animal experiments were approved by the Institutional Animal Care and Use Committee (IACUC) of the CDC and conducted in accordance with the ethical guideline for animal experiments and safety guidelines. Vaccine dosage refers to the established human dose for commercial IPV or 5 µg as full dose for IRV.

### ELISA for antigen measurements

For RV antigen measurements in IRV dMNP, we developed a RV-specific ELISA using a VP7-specific monoclonal antibody (mAb)^41^. In brief, 96-well plates were coated with rabbit anti-RV (Wa) polyclonal antibody overnight at 4 °C. The plates were washed, blocked with Superblock™ T20 (TBS) blocking buffer (Thermo Fisher Scientific) followed by incubation with a serially diluted (two-fold) solution which is reconstituted from IRV dMNP for 1 h at 37 °C. After washing, plates were added with biotin-conjugated anti-RV VP7 mAb and incubated for 1 h at 37 °C. Plates were then washed and incubated with diluted (1:10,000) Pierce™ Streptavidin Poly-HRP (Thermo Fisher Scientific) for 1 h at 37 °C and BioFX^®^ TMB One Component HRP Microwell Substrate (Surmodics Inc., MN, USA). The reaction was stopped by 1 N HCl. Plates were read with an EIA reader (Dynex Technologies, VA, USA) at dual wavelength of 450 nm and 630mn. The amount of protein in a sample was determined from a curve of purified rotavirus standard of known concentration.

For IPV, D-antigens were measured by ELISA using polio type-specific mAbs for both capture and detection as previously described^25^. Antibodies were labeled with horseradish peroxidase (HRP) using a Lightning Link Conjugation kit (HRP, 100 µg reaction kit; Novus Biologicals) to be used for antigen detection. Capture antibody solutions were prepared by adding IPV types 1, 2, or 3 (Thermo Fisher Scientific) specific antibodies to 0.05 M carbonate-bicarbonate buffer, pH 9.6. IPV type 1 and type 3 antibodies were diluted at 1:1000, and IPV type 2 antibodies were diluted at 1:500. Capture solution was added to Immulon 2HB high-binding 96-well plates (NUNC, NY, USA) and plates were incubated between > 16 h at 5 °C. Coated plates were washed with wash buffer (1x PBS; Corning, VA, USA) with 0.05% Tween 20 (Sigma-Aldrich) and incubated with blocking/dilution buffer (1x PBS with 0.5% gelatin (BD, MD, USA) and 0.25% Tween 20) for 1 h at 37 °C. After washing, antigen was added and incubated for 1 h at 37 °C. Plates were washed and detection solution, prepared by diluting the HRP-conjugated mAbs 1:1,000 in dilution buffer, was added. Plates were incubated for 1 h at 37 °C and washed. SureBlue Reserve TMB Microwell Peroxidase Substrate (1-Component) (KPL, MD, USA) was added and the reaction was allowed to proceed for approximately 15 min before the addition of TMB BlueSTOP Solution (KPL). Plates were evaluated on a SpectraMax^®^ Plus 384 microplate spectrophotometer (Molecular Devices, CA, USA) at a wavelength of 620 nm^25^.

### Quantification of IgG in rats by ELISA

Rotavirus-specific IgG in animal sera was measured using a modified enzyme immunoassay^24^. In brief, 96-well plates were coated with rabbit hyperimmune serum to RV Wa overnight at 4 °C. The plates were washed, blocked with 5% skim milk in PBS, and then incubated with supernatants of rhesus RV (RRV) (~10^6^ FFU/ml) for 1 h at 37 °C. Serial diluted (four-fold) rat serum samples were added and incubated for 1 h at 37 °C. After washing, plates were incubated with biotin-conjugated goat anti-rat IgG (Sigma-Aldrich) and Extravidin (Sigma-Aldrich) for 1 h each. TMB (Sigma-Aldrich) substrate was added for development, and the reaction was stopped with 1 N HCl. Optical density (OD_450_) was determined with an ELISA reader (Dynex Technologies). The antibody titer in serum specimen was defined as the reciprocal of the highest dilution that gave a mean OD greater than 3 standard deviations above the mean OD of the negative-serum wells.

### Microneutralization assay for RV and PV

RV-specific neutralizing activity (NA) was measured with a microneutralization assay against a homotypic RV strain, Wa (G1P[8])^42^ or a heterotypic RV strain, CDC-6 (G9P[6]) (only Post dose 3). Each strain was individually tested to optimize the amount of virus for use (700 FFU for Wa and 1,500 FFU for CDC-6 per well). Neutralizing titer was defined as the reciprocal of the highest dilution that gave a greater than 60% reduction in the absorbance (OD_450_) value compared to that in virus-only control wells.

Serum samples were tested using a standard microneutralization assay for antibodies to poliovirus types 1, 2, and 3 according to established protocols at the Global Polio Specialized Laboratory, CDC^39^. Briefly, diluted serum samples were incubated with polioviruses types 1, 2, and 3 at 35 °C for 3 h prior to addition to HEp-2(C) cells. After incubation for 5 days at 35 °C, cells were stained with crystal violet and cell viability was measured by OD_595_. Titers were determined using the Spearman–Karber method. Seropositivity was defined as antibody titers greater than or equal to 1:8.

### Statistics

All immunogenicity results were analyzed by Prism software version 7 (GraphPad, CA, USA). Comparisons among individual samples were done using an unpaired *t* test. Comparisons among multiple groups were done using a two-way ANOVA. *p* < 0.05 was considered significant.

### Reporting summary

Further information on research design is available in the Nature Research Reporting Summary linked to this article.

## Supplementary information

REPORTING SUMMARY

## Data Availability

The data that support the findings of this study are available from the corresponding author upon reasonable request.
