# Supplementary material for: Microneedle patch as a new platform to effectively deliver inactivated polio vaccine and inactivated rotavirus vaccine
Source: NPJ Vaccines. 2022 Feb 28;7:26. doi: 10.1038/s41541-022-00443-7 (PMC8885742; doi:10.1038/s41541-022-00443-7)
Supplement: Supplementary file 1 — REPORTING SUMMARY [file 41541_2022_443_MOESM1_ESM.pdf]

nature research

Corresponding author(s): Baoming Jiang

Last updated by author(s): Jan 4, 2022

Reporting Summary

Nature Research wishes to improve the reproducibility of the work that we publish. This form provides structure for consistency and transparency in reporting. For further information on Nature Research policies, see our Editorial Policies and the Editorial Policy Checklist.

Statistics

For all statistical analyses, confirm that the following items are present in the figure legend, table legend, main text, or Methods section.

n/a	Confirmed	
☐	☒	The exact sample size (*n*) for each experimental group/condition, given as a discrete number and unit of measurement
☐	☒	A statement on whether measurements were taken from distinct samples or whether the same sample was measured repeatedly
☐	☒	The statistical test(s) used AND whether they are one- or two-sided<br>*Only common tests should be described solely by name; describe more complex techniques in the Methods section.*
☒	☐	A description of all covariates tested
☒	☐	A description of any assumptions or corrections, such as tests of normality and adjustment for multiple comparisons
☐	☒	A full description of the statistical parameters including central tendency (e.g. means) or other basic estimates (e.g. regression coefficient) AND variation (e.g. standard deviation) or associated estimates of uncertainty (e.g. confidence intervals)
☒	☐	For null hypothesis testing, the test statistic (e.g. *F*, *t*, *r*) with confidence intervals, effect sizes, degrees of freedom and *P* value noted<br>*Give P values as exact values whenever suitable.*
☒	☐	For Bayesian analysis, information on the choice of priors and Markov chain Monte Carlo settings
☒	☐	For hierarchical and complex designs, identification of the appropriate level for tests and full reporting of outcomes
☒	☐	Estimates of effect sizes (e.g. Cohen's *d*, Pearson's *r*), indicating how they were calculated

Our web collection on statistics for biologists contains articles on many of the points above.

Software and code

Policy information about availability of computer code

Data collection	N/A
Data analysis	N/A

For manuscripts utilizing custom algorithms or software that are central to the research but not yet described in published literature, software must be made available to editors and reviewers. We strongly encourage code deposition in a community repository (e.g. GitHub). See the Nature Research guidelines for submitting code & software for further information.

Data

Policy information about availability of data

All manuscripts must include a data availability statement. This statement should provide the following information, where applicable:

- Accession codes, unique identifiers, or web links for publicly available datasets
- A list of figures that have associated raw data
- A description of any restrictions on data availability

N/A

Field-specific reporting

Please select the one below that is the best fit for your research. If you are not sure, read the appropriate sections before making your selection.

☒ Life sciences ☐ Behavioural & social sciences ☐ Ecological, evolutionary & environmental sciences

For a reference copy of the document with all sections, see nature.com/documents/nr-reporting-summary-flat.pdf

Life sciences study design

All studies must disclose on these points even when the disclosure is negative.

Sample size	6 animals per group is the lowest number to run and trust statistical analysis of the results or to get serum results. With a sample size of 6 animals per treatment group (a combination of vaccine and application route), a two-tailed test with the significance level of a=0.05 with a power of 80% would detect a mean difference between the treatment groups by the standard deviation times 0.44.
Data exclusions	N/A
Replication	N/A
Randomization	N/A
Blinding	N/A

Reporting for specific materials, systems and methods

We require information from authors about some types of materials, experimental systems and methods used in many studies. Here, indicate whether each material, system or method listed is relevant to your study. If you are not sure if a list item applies to your research, read the appropriate section before selecting a response.

Materials & experimental systems

n/a	Involved in the study	
☐	☒	Antibodies
☐	☒	Eukaryotic cell lines
☒	☐	Palaeontology and archaeology
☐	☒	Animals and other organisms
☒	☐	Human research participants
☒	☐	Clinical data
☒	☐	Dual use research of concern

Methods

n/a	Involved in the study	
☒	☐	ChIP-seq
☒	☐	Flow cytometry
☒	☐	MRI-based neuroimaging

Antibodies

Antibodies used	Rotavirus VP7-specific monoclonal antibody;<br>Hyperimmune sera anti-RV Wa strain;<br>monoclonal mouse antibodies specific for D-antigenic type 1, type 2, and type 3<br>Poliovirus serotype-specific (serotypes 1, 2, and 3) human sera (generated internally from poliovirus-vaccinated individuals)
Validation	Rotavirus VP7-specific monoclonal antibody(human monoclonal antibody): Nair, N. et al. Sci Transl Med 9 (2017);<br>Hyperimmune rabbit sera anti-RV Wa strain (validated internally, Jiang, B, et al. Human Vaccines 2008, 4:2, 143-147)<br>D-antigen specific antibodies: Edens C, et al. Vaccine. 2015;33(37). (IPV 1 HYB295-15-02, IPV2 HYB294-06-02, IPV3 HYB300-05-02 from Thermo Fisher Scientific)<br>Poliovirus serotype-specific (serotypes 1, 2, and 3) human sera (validated internally, drescibed in Weldon, W.C. et al. Methods Mol Biol 16 (2016)

Eukaryotic cell lines

Policy information about cell lines

Cell line source(s)	Vero, MA104, HEp-2(C) cells
Authentication	*Describe the authentication procedures for each cell line used OR declare that none of the cell lines used were authenticated.*
Mycoplasma contamination	All cell lines were confirmed negative for mycoplasma .

Commonly misidentified lines (See ICLAC register)	*Name any commonly misidentified cell lines used in the study and provide a rationale for their use.*

Animals and other organisms

Policy information about studies involving animals; ARRIVE guidelines recommended for reporting animal research

Laboratory animals	Wistar rat/ Female/ 4 weeks old
Wild animals	N/A
Field-collected samples	N/A
Ethics oversight	All animal experiments were approved by the Institutional Animal Care and Use Committee (IACUC) of the CDC and conducted in accordance with the ethical guideline for animal experiments and safety guidelines.

Note that full information on the approval of the study protocol must also be provided in the manuscript.
